# Novel perspectives on antisense transcription in HIV-1, HTLV-1, and HTLV-2

**DOI:** 10.3389/fmicb.2022.1042761

**Published:** 2022-12-23

**Authors:** Edward Lin, Amanda R. Panfil, Grace Sandel, Pooja Jain

**Affiliations:** ^1^Department of Microbiology and Immunology, Drexel University College of Medicine, Philadelphia, PA, United States; ^2^Department of Veterinary Biosciences, Center for Retrovirus Research, The Ohio State University, Columbus, OH, United States

**Keywords:** antisense transcription, HTLV-1, HIV-1, HTLV-2, MEF-2

## Abstract

The genome of retroviruses contains two promoter elements (called long terminal repeat or LTR) at the 5′ and 3′ end of their genome. Although the expression of retroviral genes generally depends on the promoter located in the 5′ LTR, the 3′ LTR also has promoter activity responsible for producing antisense transcripts. These natural antisense transcripts (NATs) are a class of RNA molecules transcribed from the opposite strand of a protein-coding gene. NATs have been identified in many prokaryotic and eukaryotic systems, as well as in human retroviruses such as human immunodeficiency virus type 1 (HIV-1) and HTLV-1/2 (human T-cell leukemia virus type 1/2). The antisense transcripts of HIV-1, HTLV-1, and HTLV-2 have been briefly characterized over the past several years. However, a complete appreciation of the role these transcripts play in the virus lifecycle and the cellular factors which regulate their transcription is still lacking. This review provides an overview of antisense transcription in human retroviruses with a specific focus on the MEF-2 family of transcription factors, the function(s) of the antisense protein products, and the application of antisense transcription models in therapeutics against HIV-1 and HTLV-1 in the context of co-infection.

## Background

Antisense-derived protein products play important roles in retroviral replication and the development of leukemia. However, few studies have been conducted on the retroviral antisense promoters and their regulation. The expression of most retroviral genes generally depends on the promoter located in the 5′ long terminal repeat (LTR). However, the 3′ LTR (antisense strand) has also been shown to have promoter activity responsible for producing a natural antisense transcript or NAT *in vivo*. NATs are RNA sequences which are transcribed from the opposite strands of DNA with partial or complete overlap with the sense RNA (Zhao et al., 2020). These NATs can be capped, polyadenylated, and undergo intron excision, just like mRNAs. In addition, the expression of NATs can be regulated by promoters and enhancers.

Derived from simian virus transmission, human T-cell lymphotropic virus type 1 (HTLV-1) is the first isolated human retrovirus responsible for pathologies in an infected individual in the form of an aggressive T-cell neoplasm adult T-cell leukemia/lymphoma (ATLL), or a neuroinflammatory disease HAM/TSP (HTLV-associated myelopathy and tropical spastic paraparesis) that has similarities to multiple sclerosis (Uchiyama et al., 1977; Poiesz et al., 1980; Yoshida et al., 1982; Gessain et al., 1985; Osame et al., 1986; Matsuura et al., 2016; Enose-Akahata et al., 2017). Sense and antisense transcription, while independent, can occur simultaneously in HTLV-1 infection. Sense transcription results in the expression of most viral genes, including the leukemogenic Tax protein. Tax acts as a transcriptional activator and is the main driver of viral transcription from the 5′ LTR (Felber et al., 1985; Yoshimura et al., 1990; Zhao and Giam, 1991). In contrast to the sporadic expression of viral transactivator protein Tax (encoded by the sense strand of the provirus and transcribed from the 5′ LTR), HBZ (HTLV-1 bZip factor) protein (encoded by the antisense strand of the provirus and transcribed from the 3′ LTR) is consistently detected in most infected cells and leukemic cells from ATLL patients (Satou et al., 2006). The degree of regulatory independence between antisense transcription and sense transcriptional activation and inhibition may have profound importance to the mechanisms of long term HTLV-1 infection, latency, and leukemogenesis (Laverdure et al., 2016). Like others cited, we too have studied the regulation of HTLV-1 molecular and infectious pathways, including regulation at the 5’ LTR by Tax (Mostoller et al., 2004; Ahuja et al., 2006, 2007; Alefantis et al., 2007; Jain et al., 2007, 2015; Manuel et al., 2009, 2013; Jaworski et al., 2014; Sagar et al., 2014).

Similar to HTLV-1/2, human immunodeficiency virus type 1 (HIV-1, the causative agent of AIDS and HIV-associated neurocognitive disorders) also encodes an antisense protein termed ASP (Affram et al., 2019). Antisense transcription has long been predicted to occur in HIV-1 due to the occurrence of an ORF in the antisense direction (Savoret et al., 2020) in group M HIV-1 strains, but not group N, O, or P strains. This indicates that it arose *de novo* with the emergence of the HIV-1 strain responsible for the global pandemic (Affram et al., 2019). This antisense reading frame is located at the junction of the gp120 and gp41 segments of the *env* gene and produces ASP, which plays a profound role in HIV-1 infection (Affram et al., 2019). ASP was commonly found in HIV-1-infected myeloid and lymphoid cell lines exclusively as an intranuclear protein (Affram et al., 2019). Contrary to predictions based on the structure of ASP, it did not integrate into the nuclear membrane, but rather localized within the nucleus in a polarized distribution and existed preferentially in areas with greater chromatin transcription. However, upon HIV-1 reactivation by PMA (phorbol 12-myristate 13-acetate), ASP protein translocated to the cytoplasm, and was found on the surface of infected cells colocalizing with gp120. In fact, in virions released from infected cells, ASP protein was found as an integral protein within the envelope of HIV-1 particles.

Retroviral coinfection has been well documented, and a growing body of evidence supports that HTLV (especially HTLV-2) and HIV-1 coinfection occurs with characteristic presentations (Beilke, 2012). The antisense protein of HTLV-2 (APH-2) maintains latency and suppresses HIV-1 replication by two mechanisms. Firstly, HIV-1 Tat-driven transactivation of transcription at the 5′ LTR is suppressed, and secondly, gag expression is decreased, preventing the release of viral particles. APH-2 over-expression inhibits HIV-1 Tat, preventing transactivation of HIV-1 LTR-driven expression of luciferase (Londhe and Kulkarni, 2021). To explain this interaction mechanistically, a terminal IXXLL motif of APH-2 was identified that was essential to the downregulatory effect (Londhe and Kulkarni, 2021). In direct contrast to the supposed function of HIV-1 antisense transcription in promoting latency and preventing reactivation, HTLV-1 antisense transcription accelerates carcinogenesis. Both the *hbz* transcript and the encoded HBZ protein have been shown to promote the survival, proliferation and tumorigenesis of HTLV-1 infected and transformed cells (Arnold et al., 2006, 2008; Mitobe et al., 2015).

## Antisense transcription in human T-cell leukemia viruses

**Figure 1:**
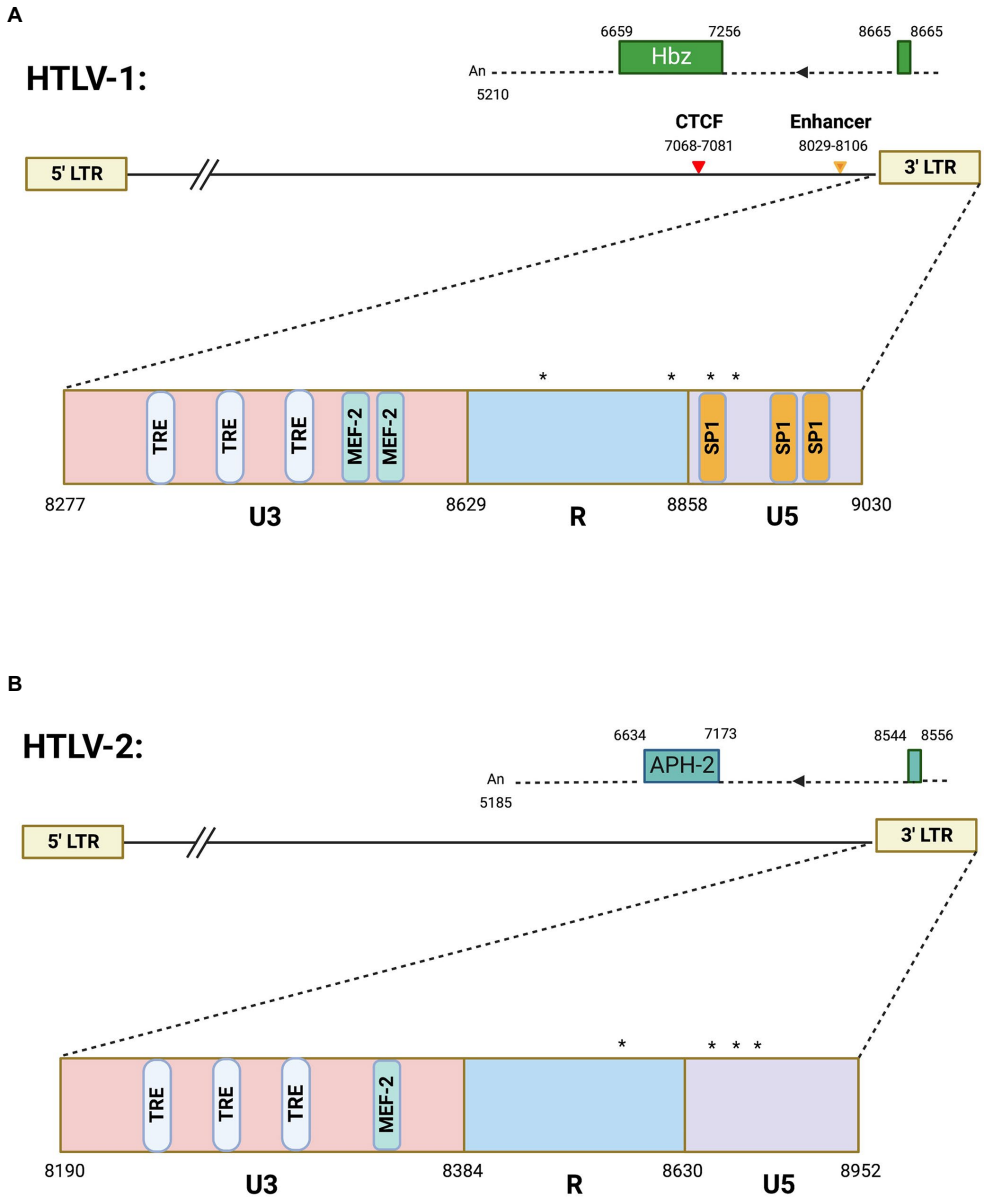
Antisense transcription in HTLV-1 and HTLV-2. Schematics of **(A)** HTLV-1 and **(B)** HTLV-2 proviral genomes highlighting the 3′ LTR region. The HTLV-1 3′ LTR contains two MEF-2 binding sites (published previously in Jain et al., 2015), several Tax responsive elements (TREs), and SP1 binding sites. Also depicted are the vCTCF binding site and Enhancer region, which overlap with the HBZ coding sequence and *hbz* intron, respectively. Spliced HBZ and APH-2 open reading frames are depicted above each proviral genome. In contrast, the HTLV-2 3′ LTR is approximately 53% identical (by sequence alignment) and contains predicted TREs and 1 potential MEF-2 binding site. Drawing is intended to be illustrative and not to exact scale. Nucleotide positions are derived from the HTLV-1 (ACH) and HTLV-2 (pH6) molecular clones. (*) denote transcription start sites. Frequently used transcription initiation sites have been identified at positions 8702, 8854, 8876, and 8882 in HTLV-1 and positions 8578, 8745, 8748, 8749, 8768, 8782, 8787, and 8788 were identified for transcription initiation sites in HTLV-2.

HTLV-1 antisense transcription produces the antisense protein HBZ. There are two isoforms of the HBZ protein that arise from a spliced and unspliced version of the *hbz* transcript (Cavanagh et al., 2006). Both the spliced and unspliced *hbz* mRNAs have TATA-less promoters, while the spliced *HBZ* gene is regulated by SP1 (Yoshida et al., 2008). The HBZ protein which arises from the spliced transcript is more abundant in cells and has stronger cellular effects than unspliced HBZ (Yoshida et al., 2008). Nearly all studies within the HTLV-1 field have focused on the spliced *hbz* mRNA and thus all reference to *hbz* mRNA transcript in this review is referencing spliced RNA. Several different *hbz* transcriptional start sites have been mapped within the HTLV-1 genome (Cavanagh et al., 2006). The most frequently used sites are highlighted in Figure 1A. Multiple transcript initiation sites are thought to arise from the lack of a TATA box at close distance. A previous study found that both spliced and unspliced *hbz* transcripts were predominantly localized to the nucleus in both provirus transfected HLtat cells and the C91PL cell line (chronically infected T-cell line; Rende et al., 2011). A recent study found that the HTLV-1 3′ LTR induces poor polyadenylation (shorter than average polyA tails) that results in the nuclear retention of *hbz* transcript (Ma et al., 2021b).

Simultaneous sense and antisense transcription raise the possibility that the gene body is occupied and clogged by polymerases. A study by Laverdure et al. showed that transcriptional regulation of the HTLV-1 3′ LTR is surprisingly independent of sense transcription (Laverdure et al., 2016). This suggests bidirectional transcription would not restrict *hbz* or *tax* expression. Interestingly, this study found a population of cells that expressed Tax protein and had detectable antisense transcription but lacked sense transcription. Upon further evaluation, this population of cells was arrested in the G0/G1 phase of the cell cycle. Taken together, these results demonstrated the effect of Tax on cell cycle arrest can inhibit its continued function as a sense transcriptional activator, but it does not affect continued antisense transcription.

While Tax expression is considered to be the primary driver of transformation and oncogenesis, this protein is actually lost in 60% of ATLL cases indicating that the persistence of Tax is not necessary for the maintenance of ATLL (Zhao and Matsuoka, 2012). In contrast, *hbz* transcript encoded by the 3′ LTR is more consistently detected in infected cells, asymptomatic HTLV-1 carriers, and ATLL patient samples (Satou et al., 2006; Landry et al., 2009). The few studies conducted on HTLV-1 antisense promoters have placed great importance on the function of HBZ as a regulator of carcinogenesis *via* dysregulation of telomerase in infected cells (Borowiak et al., 2013). HBZ promotes proliferation of T cells, increasing G1/S transition (Satou et al., 2006). The HBZ protein contains three domains: an N-terminal activation domain, central domain, and basic ZIP domain (Zhao and Matsuoka, 2012). Within the HBZ N-terminal domain are motifs that promote binding to p300/CBP. This binding acts as a regulator for a number of cellular processes including proliferation, cell cycle regulation, apoptosis, differentiation, and responding to DNA damage (Iyer et al., 2004; Zhao and Matsuoka, 2012). p300/CBP is also implicated in several cancers since somatic mutations are found in a number of malignancies and chromosomal translocations which target p300/CBP have been found in acute myeloid leukemia and treatment-related hematological disorders (Iyer et al., 2004). The HBZ protein has been shown to interact with several cellular transcription factors such as JunD (Thebault et al., 2004), JunB, and c-Jun (Basbous et al., 2003; Matsumoto et al., 2005), and is a negative regulator of Tax-mediated HTLV-1 transcription (Gaudray et al., 2002). Importantly, HBZ protein is required for enhanced viral infectivity and persistence in inoculated rabbits (Arnold et al., 2006), and it promotes cell proliferation and tumor cell growth in cell culture and a murine transplantation model (Arnold et al., 2008). Also, HBZ is tumorigenic in transgenic mice (Satou et al., 2011). Several groups have shown evidence the *hbz* mRNA supports proliferation of ATL tumor cells (Satou et al., 2006; Mitobe et al., 2015) suggesting the gene has bimodal functions in two different molecular forms (mRNA and protein). This is rare, but not unique, as mRNA and corresponding protein of some cellular genes also serve multiple functions in cells (i.e., p53, VEGF, and HMGA2; Candeias et al., 2008; Masuda et al., 2008; Kumar et al., 2014).

HTLV-2 is a closely related retrovirus to HTLV-1. While both viruses encode proteins with similar structure, they differ in pathogenicity (Ciminale et al., 2014). HTLV-2 is not strongly associated with disease like HTLV-1. HTLV-2 antisense transcription produces the antisense protein APH-2 (antisense protein of HTLV-2). Unlike *hbz* mRNA, no alternative splicing has been observed for the *aph-2* transcript (Halin et al., 2009). The experimentally identified *aph-2* transcriptional start sites are denoted in Figure 1B. Like *hbz*, the *aph-2* transcript has several transcription initiation sites likely arising from the lack of a TATA box (Halin et al., 2009). HBZ and APH-2 have been shown to share similar functions, such as inhibition of Tax-mediated transcription (Halin et al., 2009). However, these antisense derived proteins also have divergent roles in viral pathobiology. Loss of APH-2 increases HTLV-2 replication and proviral load in a rabbit model of infection (Yin et al., 2012), while loss of HBZ decreases HTLV-1 replication and proviral load (Arnold et al., 2006). Figure 1 provides an overview of antisense transcripts in HTLV-1 and -2. Comparative studies between these similar, yet pathogenically distinct, retroviruses will provide a better understanding of the role of antisense proteins in disease development and maintenance.

DNA elements within the HTLV-1 provirus, including a viral enhancer (Matsuo et al., 2022) and the insulator region containing a CTCF-binding site (CTCF-BS; Satou et al., 2016), have been shown to have roles in the regulation of sense versus antisense viral gene expression. CTCF is a key regulator of chromatin structure and function. The viral CTCF-BS (vCTCF-BS) is located at a sharp border in epigenetic modifications within the pX region of the proviral genome (Satou et al., 2016). The presence of the vCTCF-BS disrupts host chromatin structure and looping, regulates viral mRNA splicing, and maintains/regulates the epigenetic barrier found in the pX region (Melamed et al., 2018; Cheng et al., 2021). However, loss of the vCTCF-BS did not disrupt the ability of the virus to immortalize target cells *in vitro* or establish persistence in a rabbit model of infection (Martinez et al., 2019). Quite recently, an enhancer element was identified within the HTLV-1 genome through a screen for nucleosome free regions (Matsuo et al., 2022). Cellular transcription factors SRF (serum responsive factor) and ELK-1 (ETS Like-1 protein) were found to bind at this region. Together these form a ternary complex at multiple serum response elements. Ablation of these SRF/ELK-1 binding sites decreased enhancer activity in transfected cells (Satou et al., 2011). The loss of the enhancer element did not affect the ability of HTLV-1 to establish persistence in the rabbit model of infection, induce disease in humanized mice, or immortalize cells *in vitro* (Maksimova et al., 2022). However, it did alter the cellular immortalization phenotype (shift from predominantly CD4^+^ to CD8^+^ T cells), decreased proviral load, and decreased gene expression. Together, these results suggest that the enhancer element alone does not determine persistence and disease development but plays a critical role in regulating viral gene expression in the infected cell.

Additional insight on the regulation of HTLV-1 antisense promoter comes from recent studies from authors’ laboratories on a unique transcription factor called Myocyte Enhancer Factor (MEF)-2 that was previously established to have a role in HTLV-1 pathogenesis (Jain et al., 2015). More recent studies investigated all four MEF-2 isoforms (A-D, reviewed in (Madugula et al., 2022)), of which two (MEF-2A and -2C) were highly overexpressed in a wide array of HTLV-1 infected and ATLL cell lines as well as in acute ATLL patients (Madugula et al., 2022). Knockdown of these isoforms led to decreased cell proliferation and regulated cell cycle progression. Chemical inhibition of MEF-2 resulted in the cytotoxicity of ATLL cells *in vitro* and reduction of proviral load in a humanized mouse model. These studies provided a novel mechanism of 3′ LTR regulation and established MEF-2 signaling as a potential target for therapeutic intervention for ATLL. MEF-2C was found to be highly enriched at the 3′ LTR along with cofactors Menin and JunD resulting in binding of MEF-2C to HBZ at this region. JunD is critical for the regulation of both HIV-1 and HTLV-1 3′ LTR (Gazon et al., 2017), and upregulates the transcription of hTERT catalytic subunit of human telomerase. In normal cells, JunD is inhibited by a tumor suppressor (the product of the MEN-1 gene) and thus hTERT, along with functional telomerase levels are downregulated (Borowiak et al., 2013). MEF-2C exhibits strong binding with Menin, thus liberating JunD from its inhibitory clutch. Since there is a well-established link between HBZ/JunD and telomerase upregulation, future studies should explore the role of MEF-2C in JunD-mediated regulation of hTERT and telomerase in the context of HTLV-1-induced immortalization and carcinogenesis of T cells. The HTLV-1 3′ LTR contains two MEF-2 binding sites as published before (Jain et al., 2015), several Tax responsive elements (TREs), and SP1 binding sites depicted in Figure 1. In contrast, the HTLV-2 3′ LTR, which is approximately 53% identical by sequence alignment, contains predicted TREs and 1 potential MEF-2 binding site (Figure 1) that needs to be experimentally validated.

Typical expression of MEF-2 isoforms occurs in almost every human tissue, ranging from hematopoietic stem cells to the cerebral cortex. These MEF-2 isoforms play key roles in controlling cellular differentiation and maintenance of mature cell types. MEF-2 isoforms, mainly MEF-2A and -2C, have also been implicated in various leukemias and lymphomas, as well as virus infection (reviewed in Madugula et al., 2019). MEF-2C has been shown to be involved in Epstein–Barr virus (EBV), which is a causative agent of Burkitt’s lymphoma, Hodgkin’s lymphoma, CNS lymphoma, gastric carcinoma, and nasopharyngeal carcinoma. During EBV infection, certain clusters of enhancers are marked by a high degree of H3K27ac chromatin immunoprecipitation enrichment, demonstrating a great deal of epigenetic control (Wang et al., 2019). MEF-2C is one of the many transcription factors necessary for the proliferation of EBV-induced lymphoblastoid cell lines (Wang et al., 2019). The disruption of transcription factor binding to the super enhancer regions was able to disrupt EBV related oncogenic transformation and proliferation (Wang et al., 2019). Hence, the combined dysregulation of transcription factors including MEF-2C at clusters of enhancers can lead to the oncogenic state seen in EBV infection. Considering that this same pattern of MEF-2C binding at the HTLV-1 3′ LTR is seen in ATLL oncogenesis, continued investigation of the function of MEF-2 isoforms in viral tumorigenesis is necessary, especially as it relates to the control of antisense transcription.

Chromatin integration could influence 3′ LTR transcription (Landry et al., 2009; Barbeau et al., 2013). Previous studies in both latently infected cell lines and ATL cells found selective hypermethylation of CpG sites within the 5′ LTR and hypomethylation within the 3′ LTR using bisulfite sequencing (Koiwa et al., 2002). The fact that the 3′ LTR is not methylated is interesting. Previously higher levels of di- and tri-methylated lysine 4 of histone H3 (H3K4me2 or H3K4me3) were found at the 3′ LTR compared to the 5′ LTR in HTLV-1-infected cells (Lemasson et al., 2004). It is possible that one or more members of the KMT2 methyl-transferase family, which are responsible for these transcriptionally active modifications, contribute to HBZ expression. This mode of regulation could help explain how the antisense promoter is consistently maintained in an unmethylated, active state in ATL cells. H3K4me2 and H3K4me3 potentially inhibit *de novo* DNA methylation by preventing H3K9 methylation within the same region (H3K9 methylation is a repressive modification that may serve as a molecular beacon for certain DNA methyltransferases). Interestingly, MEF-2C has been shown to interact with the chromatin architecture of schizophrenia and improves cognition in mice (Mitchell et al., 2018). MEF-2C may also influence the epigenetics of the antisense region of HIV-1 and HTLV-1 genomes, which should be explored in future studies.

## Antisense transcription in human immunodeficiency virus type I

An antisense transcript, termed *asp,* has been detected using RT-PCR analysis in HIV-1-infected cells (Landry et al., 2007). Like HTLV-1 and HTLV-2, the HIV-1 antisense transcript also initiates in the 3′ LTR from a TATA-less promoter that functions independently of the viral transcriptional activator Tat. Several different groups have identified multiple transcriptional start sites with variable transcript length for *asp* (Li et al., 2021), however each transcript encodes for a functional protein called ASP. Similar to HTLV-1 *hbz* transcript, the HIV-1 3′ LTR produces an *asp* transcript with a poor polyadenylation site and therefore the transcript is predominantly nuclear (Ma et al., 2021b). Current models of HIV-1 sense and antisense transcription propose that the two directionalities operate antagonistically. This model reconciles the potential for transcriptional interference that concomitant sense and antisense transcription would have on each other, and seems to be supported in that productively infected cells predominantly transcribe in the sense direction; meanwhile, antisense transcriptional activity seems to curtail the reactivation of latent HIV-1 (Kobayashi-Ishihara et al., 2018; Savoret et al., 2020). While sense mRNA could only be detected in 10% of HIV-1 latently infected cell lines, over 80% of these cell lines overexpressed *asp* RNA levels, further indicating the negative correlation and antagonistic relationship between sense and antisense HIV-1 RNA transcriptions (Ma et al., 2021a). HIV-1 antisense transcription may also yield endogenous long non-coding RNA (lncRNA) strands that can act as transcriptional regulators at the 5′ LTR, epigenetically regulating viral latency (Saayman et al., 2014). Using small RNAs (smRNAs) targeted to lncRNA regions on the antisense strand will downregulate lncRNAs, leading to activation of the HIV-1 virus (Saayman et al., 2014).

**Figure 2:**
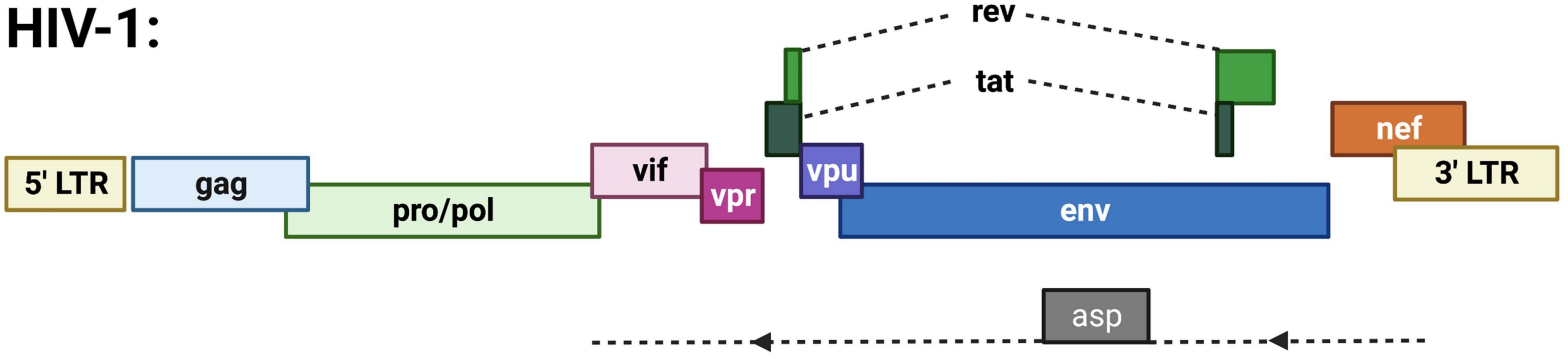
Schematic of the HIV-1 proviral genome highlighting known ORFs. Depicted are the gag, pro/pol, and env ORFs encoding the structural and enzymatic genes. Regulatory/accessory proteins encoded from the sense strand of the genome include Rev., Tat, Vif, Vpr, Vpu, and Nef. The only anti-sense derived protein is ASP.

HIV-1 antisense RNA is primarily localized to the nucleus, with less polyadenylation than HIV-1 sense RNAs (Ma et al., 2021a). Antisense transcription of the HIV-1 genome (outlined in Figure 2) produces an antisense protein called ASP. This protein is functional and becomes integrated into the HIV-1 viral structure (Affram et al., 2019). ASP localizes with the cell membrane of viral particles and infected cells, close to the gp120 envelope glycoprotein (Affram et al., 2019). It is suggested that ASP promotes viral replication since cell membrane and cytoplasmic localization of ASP is correlated with maximal HIV-1 expression (Affram et al., 2019). Recent studies have demonstrated the role of ASP in autophagy, which is necessary for developmental processes, cellular stress responses, and immune pathways induced by pathogens (Liu et al., 2019). Autophagy involves the degradation of proteins and organelles present in a cell (Torresilla et al., 2013). Varying levels of autophagy are induced depending on the specific ASP clade (A, B, C, D, or G) that functions in association with p62, a ubiquitin sensor and adaptor protein (Liu et al., 2019). Although the specific mechanism of autophagy induction remains unclear, it is because of the role of ASP in autophagy that intracellular levels of the antisense protein are hard to detect (Torresilla et al., 2013). However, with the knowledge of ASP location and functionality, it is possible to exploit this protein for therapeutic effects in HIV-1 patients (Affram et al., 2019).

## Antisense transcription models in retroviral therapeutics

Regarding the application of new therapeutic technologies, much discussion has been raised surrounding the use of CRISPR (Clustered Regularly Interspaced Short Palindromic Repeats) for antiretroviral therapy (Panfil et al., 2020). Prior to the use of CRISPR systems, zinc finger nucleases (ZFNs) targeting LTR promoters were utilized to inhibit the proliferation of HTLV-1 infected cell lines (Tanaka et al., 2013). In this methodology published in 2013, the therapeutic effects of HTLV-1 elimination were supported *in vivo*, and it was further suggested that zinc finger nucleases could be used to remove HTLV-1 proviral genomes from infected cells for therapeutic benefit (Tanaka et al., 2013). However, with the onset of wider application of CRISPR technologies, multiple factors suggest that CRISPR may be a more optimized alternative to ZFN-based therapies, including greater cost-effectiveness, simplicity, and efficiency (Panfil et al., 2020). Even greater support lies in that such implementation has already been shown to effectively decrease ATLL cell proliferation *in vitro* (Nakagawa et al., 2018). Two gRNAs targeting HBZ, the product of 3′ LTR antisense transcription, were demonstrated to be toxic to ATLL cell lines, patient samples, and xenografts. Further study of the application of CRISPR technologies should seek to demonstrate benefit *in vivo*, using animal models.

Therapeutic applications of CRISPR technology extend to HIV-1 as well. For some time, the concept of using CRISPR gene editing as a therapeutic intervention for HIV-1 infection has been considered as a key research point. While highly active anti-retroviral therapy (HAART) drugs have remained the mainstay of HIV-1 treatment, these drugs act by suppressing HIV-1 replication, and do not effectively eradicate HIV-1 proviral DNA from latently infected cells. Once HAART therapy is ceased, the latent infection is able to rebound and continue to debilitate patients (Eggleton and Nagalli, 2022; Maslennikova and Mazurov, 2022). Thus, the clear challenge in the race for an effective cure for HIV-1 infection lies in the removal of proviral DNA in latently infected cells, a similar challenge to that of HTLV-1 infection, and in fact all retroviral infections. Multiple strategies exist for anti-HIV-1 applications of CRISPR, and they can be generally grouped into knock-out and knock-in strategies (Maslennikova and Mazurov, 2022). Knock-out of viral coreceptors CCR5 and CXCR4 and direct targeting of active and inactive proviral DNA exist as two strategies targeting the removal of critical stages of HIV-1 infection (Yu et al., 2018). Meanwhile, knock-in strategies seek to introduce therapeutic genes or introduce mutations in viral restriction factors that can target HIV-1. All these strategies seek a state of a “functional cure” in which even with minimal HAART dosing, the virus cannot replicate. These strategies, however, are not without drawbacks ranging from the difficulty in knock-out selection due to low expression, as in the case of the CCR5 co-receptor, to lack of effectiveness against different tropisms and possibility of mutant escape. Off-target effects are also still a considerable drawback to all CRISPR therapeutic approaches. Much work is still needed to perfect these models for CRISPR therapeutics for the cure of HIV-1, and it is likely that HAART will remain first line for a considerable time (Maslennikova and Mazurov, 2022).

## Conclusions and perspective

Retroviruses such as HIV-1 and HTLV-1 contain LTRs at both ends of their genome. While it is well established that retroviral gene expression is dependent mainly on the 5′ LTR, antisense transcription originating from the 3′ LTR produces mRNA and protein products that have a great deal of influence on the course of retroviral infection in both HIV-1 and HTLV-1 (summarized in Table 1). In fact, the HTLV-1 antisense transcript has been demonstrated to play vital roles in the retroviral replication cycle. Furthermore, the induction of oncogenesis and ATLL from HTLV-1 infection is greatly dependent on antisense transcription and expression of the antisense protein HBZ. While the sense product, Tax, is lost or intermittently silenced in 60% of ATLL cases, HBZ is consistently detected and necessary for development of ATLL. Meanwhile, the HIV-1 antisense protein ASP has also been shown to be expressed both within the nucleus of infected cells, and on the surface of HIV-1 virions as an integral protein. The regulation of these antisense transcripts may largely be controlled by MEF-2 transcription factor isoforms, as overexpression of the MEF-2A and -2C isoforms were demonstrated in HTLV-1 induced ATLL. MEF-2 knockdown experiments demonstrated ATLL toxicity *in vitro* and decreased proviral load *in vivo*. MEF-2C also plays a role in several other leukemias and lymphomas. Notably, it plays a role in EBV oncogenesis and in the proliferation of EBV-induced lymphoblastoid cell lines. Disruption of MEF-2C and other transcription factors binding to “super enhancer” regions disrupts EBV oncogenesis.

**Table 1:** Human retroviral antisense transcription.

Retrovirus	HTLV-1	HTLV-2	HIV-1
*Antisense transcript*	*hbz*	*aph-2*	*asp*
*Overlaps with sense RNAs*	Yes	Yes	Yes
*Transcription start site(s)*	Multiple start sites in 3′ LTR, lacks a TATA box	Multiple start sites in 3′ LTR, lacks a TATA box	Multiple start sites in 3′ LTR, lacks a TATA box
*Transcription factor regulation*	MEF-2C (Menin/JunD also), SP1, viral enhancer (SRF, ELK-1)	SP1	SP1
*Spliced transcript*	Yes, spliced transcript is more abundant	Yes	No
*Cellular localization*	Predominantly nuclear due to poor polyadenylation	Unknown	Predominantly nuclear due to poor polyadenylation
*Known function*	*hbz* transcript supports proliferation, HBZ protein suppresses Tax transcription, promotes tumorigenesis	APH-2 protein suppresses Tax-2 transcription	Translated ASP protein is integrated into the membrane of budding virions, promotes viral replication, promotes latency and prevents viral reactivation, role in autophagy?
*Bidirectional transcription*	Yes	Unknown	Not detected

A possible avenue of investigation and therapy exists with CRISPR technology. There is potential in both identifying transcription factors that bind to the 3′ LTR in HTLV-1, as well as in a wider set of therapeutic applications involving knock-out of key genes for viral proliferation, or knock-in of therapeutic genes or gene mutations. The understanding of antisense transcription in retroviruses is still in its infancy. Much remains to be discovered regarding the roles of antisense transcripts and their protein products in the regulation of retroviral life cycles, as well as virally induced oncogenesis. There is a potential for new therapeutic strategies against both retroviral infection as well as their oncogenic endpoints.

## Author contributions

EL and AP equally contributed to writing of the manuscript. GS contributed to writing of the manuscript. PJ conceived the idea, assisted with writing of the manuscript, edited text, and figure drafts. All authors contributed to the article and approved the submitted version.

## Funding

Authors acknowledge funding support from the NIH/NINDS *via* R01 NS097147 to PJ.

## Conflict of interest

The authors declare that the research was conducted in the absence of any commercial or financial relationships that could be construed as a potential conflict of interest.

## Publisher’s note

All claims expressed in this article are solely those of the authors and do not necessarily represent those of their affiliated organizations, or those of the publisher, the editors and the reviewers. Any product that may be evaluated in this article, or claim that may be made by its manufacturer, is not guaranteed or endorsed by the publisher.
